# Programmed death ligand-1 expression in adrenocortical carcinoma: an exploratory biomarker study

**DOI:** 10.1186/s40425-015-0047-3

**Published:** 2015-02-17

**Authors:** André P Fay, Sabina Signoretti, Marcella Callea, Gabriela H Telό, Rana R McKay, Jiaxi Song, Ingrid Carvo, Megan E Lampron, Marina D Kaymakcalan, Carlos E Poli-de-Figueiredo, Joaquim Bellmunt, F Stephen Hodi, Gordon J Freeman, Aymen Elfiky, Toni K Choueiri

**Affiliations:** Dana-Farber Cancer Institute, 450 Brookline Avenue (DANA 1230), Boston, MA 02215 USA; Programa de Pós-Graduação em Medicina e Ciências da Saúde, Faculdade de Medicina, Pontifícia Universidade Católica do Rio Grande do Sul, Av. Ipiranga 6690, Porto Alegre, RS 90619-900 Brazil; Brigham and Women’s Hospital, 75 Francis Street (Thorn Building 504A), Boston, MA 02215 USA; Harvard Medical School, 25 Shattuck Street, Boston, MA 02115 USA; Joslin Diabetes Center, One Joslin Place, Boston, MA 02215 USA; Center of Immuno-Oncology, 450 Brookline Avenue, Boston, MA 02215 USA

**Keywords:** Adrenocortical carcinoma, PD-L1, PD-1 inhibitors, Immunotherapy

## Abstract

**Background:**

Adrenocortical carcinoma (ACC) is a rare tumor in which prognostic factors are still not well established. Programmed Death Ligand-1 (PD-L1) expression in ACC and its association with clinico-pathological features and survival outcomes are unknown.

**Methods:**

Formalin-fixed paraffin-embedded (FFPE) specimens were obtained from 28 patients with ACC. PD-L1 expression was evaluated by immunohistochemistry (IHC) in both tumor cell membrane and tumor infiltrating mononuclear cells (TIMC). PD-L1 positivity on tumor cells was defined as ≥5% tumor cell membrane staining. TIMC were evaluated by IHC using a CD45 monoclonal antibody. For PD-L1 expression in TIMC, a combined score based on the extent of infiltrates and percentage of positive cells was developed. Any score greater that zero was considered PD-L1 positive. Baseline clinico-pathological characteristics and follow up data were retrospectively collected. Comparisons between PD-L1 expression and clinico-pathological features were evaluated using unpaired t-test and Fisher’s exact test. Kaplan-Meier method and log-rank test were used to assess association between PD-L1 expression and 5-year overall survival (OS).

**Results:**

Among 28 patients with surgically treated ACC, 3 (10.7%) were considered PD-L1 positive on tumor cell membrane. On the other hand, PD-L1 expression in TIMC was performed in 27 specimens and PD-L1 positive staining was observed in 19 (70.4%) patients. PD-L1 positivity in either tumor cell membrane or TIMC was not significantly associated with higher stage at diagnosis, higher tumor grade, excessive hormone secretion, or OS.

**Conclusions:**

PD-L1 expression can exist in ACC in both tumor cell membrane and TIMC with no relationship to clinico-pathologic parameters or survival.

**Electronic supplementary material:**

The online version of this article (doi:10.1186/s40425-015-0047-3) contains supplementary material, which is available to authorized users.

## Background

Adrenocortical carcinoma (ACC) is a rare and highly lethal malignancy arising from the adrenal cortex. In the United States, around 0.7-2 new cases per million are estimated every year [1,2]. Overall, ACC carries a poor prognosis with the most consistent prognostic factor being tumor stage at the time of diagnosis [3]. Unfortunately, retrospective studies have reported a 5-year survival rate of 24% for stage III and 0% for stage IV disease [4].

Complete surgical resection remains the only chance of cure for patients with early-stage disease (stage I and II). When feasible, resection is the mainstay of therapy, even for patients with locally advanced disease. Not without controversy, some studies suggest that adjuvant mitotane may improve clinical outcomes [5]. However, despite aggressive management, close to 80% of surgically treated patients will develop subsequent metastatic disease [6].

The management of metastatic disease is challenging and disappointing results have been reported with the few available systemic therapeutic options [7]. Despite no statistically significant impact on overall survival (OS), results from the First International Randomized Trial in Locally Advanced and Metastatic ACC Treatment (FIRM-ACT) study have provided most consistent evidence for systemic treatment in advanced ACC [8]. This collaborative effort evaluated two different widely recommended regimens based on small phase II clinical trials [9,10]: mitotane plus a combination of etoposide, doxorubicin, and cisplatin (M/EDP) or mitotane plus streptozocin (SM). Patients who were treated with M/EDP had a significantly longer progression-free survival (PFS) compared with those who received SM (5.0 vs 2.1 months). Subsequent systemic options after progression on M/EDP or SM include gemcitabine plus capecitabine or metronomic 5-fluouracil which showed some clinical activity in a phase II clinical trial [11].

The biology underlying ACC is poorly understood [12]. So far, small studies have failed to demonstrate clinical benefit with targeted therapies blocking the epidermal growth factor receptor (EGFR), vascular endothelial growth factor (VEGF), mammalian target of rapamycin (mTOR), insulin-like growth factor 1 receptor (IGF-1R), or fibroblast growth factor receptor (FDFR) pathways in advanced disease, and no biomarkers have been established as predictors of survival or response to these agents [13-18]. The Tumor Cancer Genome Atlas is ongoing for ACC. This initiative will provide a comprehensive molecular characterization of this disease and may help to identify targets for drug development or a prognostic signature for risk stratification.

The current progress in understanding how the immune system can modulate tumor progression or effective responses against cancer is unfolding [19]. Immune checkpoints, like programmed death-1 (PD-1) and its ligand PD-L1, have been described as key regulators of T cell responses, and blocking the PD-1/PD-L1 axis using monoclonal antibodies has resulted in promising results in different malignancies [20]. Notably, in some series, levels of PD-L1 expression have correlated with clinical outcome [21,22]. However, the prognostic impact of PD-L1 expression still needs to be defined in many tumor types including ACC.

In this study, our goal is to characterize PD-L1 expression in ACC tissues and to correlate levels of PD-L1 expression with clinico-pathological features as well as survival outcomes.

## Results

### Patients and tumor characteristics

A total of 28 patients with ACC were included in this study. Patient characteristics are summarized in Table 1. Overall, the median age was 47.4 +/−13.2 years ranging from 19.8 to 73.7 years. Mean tumor size was 10.9 +/−4.4 cm ranging from 2.5 to 19 cm. The stage at diagnosis was defined pathologically according to International Union Against Cancer (UICC) and European Network for the Study of Adrenal Tumors (ENSAT) staging systems [23]. UICC stage I, II, III and IV were found in 1, 9, 3 and 11 patients, respectively, and ENSAT stage I, II, III, IV were found in 1, 9, 4 and 10 patients, respectively. Additionally, 19 patients developed metastasis during the follow up period and 14 patients presented with functional tumors at diagnosis.

**Table 1:** **Patient characteristics**

**Characteristics**		**Total (N = 28)**	
		**No. of patients**	**%**
**Sex**	Male	13	46.4
	Female	15	53.6
**Stage at Diagnosis (UICC)**	I	1	4.2
	II	9	37.5
	III	3	12.5
	IV	11	45.8
	Missing	4	*-*
**Stage at Diagnosis (ENSAT)**	I	1	*4.2*
	II	9	*37.5*
	III	4	*16.6*
	IV	10	*41.7*
	Missing	4	*-*
**Grade**	Low	1	8.3
	High	11	91.7
	Missing	16	-
**Metastatic disease**	Yes	19	67.9
	No	9	32.1
**Sites of Metastasis**	Local Recurrence	4	14.3
	Lymph Node	8	28.6
	Lung	10	35.7
	Liver	8	28.6
	Bone	2	7.1
	Other	3	10.7
**Functional Tumors at Diagnosis**	Yes	14	50
	No	14	50
**PD-L1 Expression on Tumor Cell Membrane**	<5% (negative)	**25**	***89.3***
	≥5% (positive)	**3**	***10.7***
PD-L1 Expression in Tumor Infiltrating Mononuclear Cells (TIMC)*	Score = 0 (negative)	**8**	***29.6***
	Score > 0 (positive)	**19**	***70.4***
		**Mean +/− SD**	**Min, Max**
**Age at Diagnosis (years)**		47.4 +/−13.2	19.8 - 73.7
**Tumor size (cm)**		10.9 +/−4.4	2.5 -19
**Mitosis/10HPF**		13.7 +/−13.5	0.2-50

*In 1 patient, the PD-L1 staining in TIMC was not assessable.

In this cohort, 8 specimens were from metastasis and 20 from primary tumors. Among the metastatic specimens, only one had metastatic disease at diagnosis. Others had specimens collected at the time of relapse.

### Correlation of PD-L1 expression and clinico-pathological features

**Figure 1:**
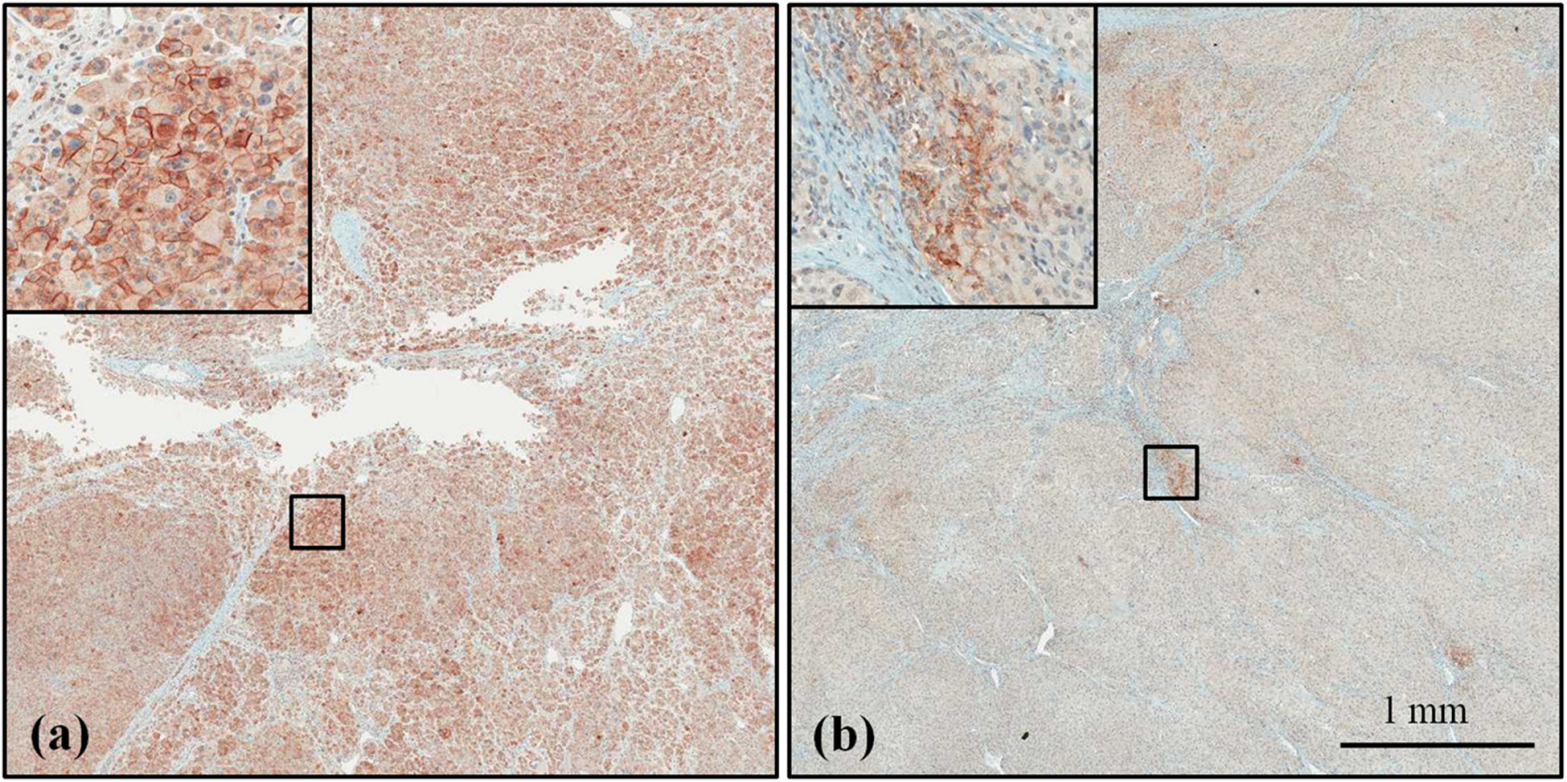
**PD-L1 Expression in FFPE ACC Samples Stained with Anti-PD-L1 Antibody (clone 405.9A11).** Positive membranous staining is present in tumor cells in panel **(a)**. In panel **(b)**, tumor cells are negative, and TIMC are positive for PD-L1.

Overall, PD-L1 expression on tumor cell membrane was considered positive in 3 of 28 patients (10.7%) (Figure 1). On univiariate analysis, PD-L1 expression was not correlated with stage (UICC and/or ENSAT), grade, or excessive secretion of hormones (Table 2).

**Table 2:** **Patient characteristics according to PD-L1 expression on tumor cell membrane**

**Characteristics (N = 28)**	**% Positive tumor cell membrane**			
	**<5% (negative) (n = 25, 89.3%) n(%)**	**5% or more (positive) (n = 3, 10.7%) n(%)**		P-value*
UICC Stage**	I/II	9(90)	1(10)	-
	III/IV	13(92.8)	1(7.2)	
ENSAT Stage**	I/II	9(90)	1(10)	-
	III/IV	13(92.8)	1(7.2)	
Grade***	Low	1(100)	0(0)	-
	High	10(90.9)	1(9.1)	
**Functional Tumors at Diagnosis**	Yes	14(100)	0(0)	0.22
	No	11(78.6)	3(21.4)	

*Fisher’s Exact Test.

**Missing: 4.

***Missing: 12.

A total of 27 patients were evaluated for PD-L1 expression in tumor infiltrating mononuclear cells (TIMC). The extent of TIMC were recorded as focal in 7 patients (26%), mild in 9 patients (33.3%), moderate in 9 patients (33.3%), and high in 2 patients (7.4%). For PD-L1 expression in TIMC, scores greater than zero were identified in 19 patients (70.4%). There was no significant correlation between PD-L1 expression in TIMC and stage (UICC and/or ENSAT), grade, or excessive hormone secretion (Table 3).

**Table 3:** **Patient characteristics according to PD-L1 expression on TIMC**

**Characteristics (n = 27)**		**Tumor infiltrating mononuclear cells**		
		**Score = 0 (negative) (n = 8, 29.6%) n(%)**	**Score > 0 (positive) (n = 19, 70.4%) n(%)**	P-value*
UICC Stage**	I/II	1(11.1)	8(88.9)	0.34
	III/IV	5(35.7)	9(64.3)	
ENSAT Stage**	I/II	1(11.1)	8(88.9)	0.34
	III/IV	5(35.7)	9(64.3)	
Grade***	Low	0(0)	1(100)	-
	High	3(30)	7(70)	
**Functional Tumors at Diagnosis**	Yes	4(30.8)	9(69.2)	-
	No	4(28.6)	10(71.4)	

*Fisher’s Exact Test.

**Missing: 4.

***Missing: 16.

We further explored the effect of PD-L1 expression over other variables such as site of metastasis, number of mitosis per 10 high-power fields, age or tumor size. However, no association was found between any of these parameters and PD-L1 expression in either tumor cell membrane or TIMC (data not shown).

### Correlation of PD-L1 expression and overall survival

**Figure 2:**
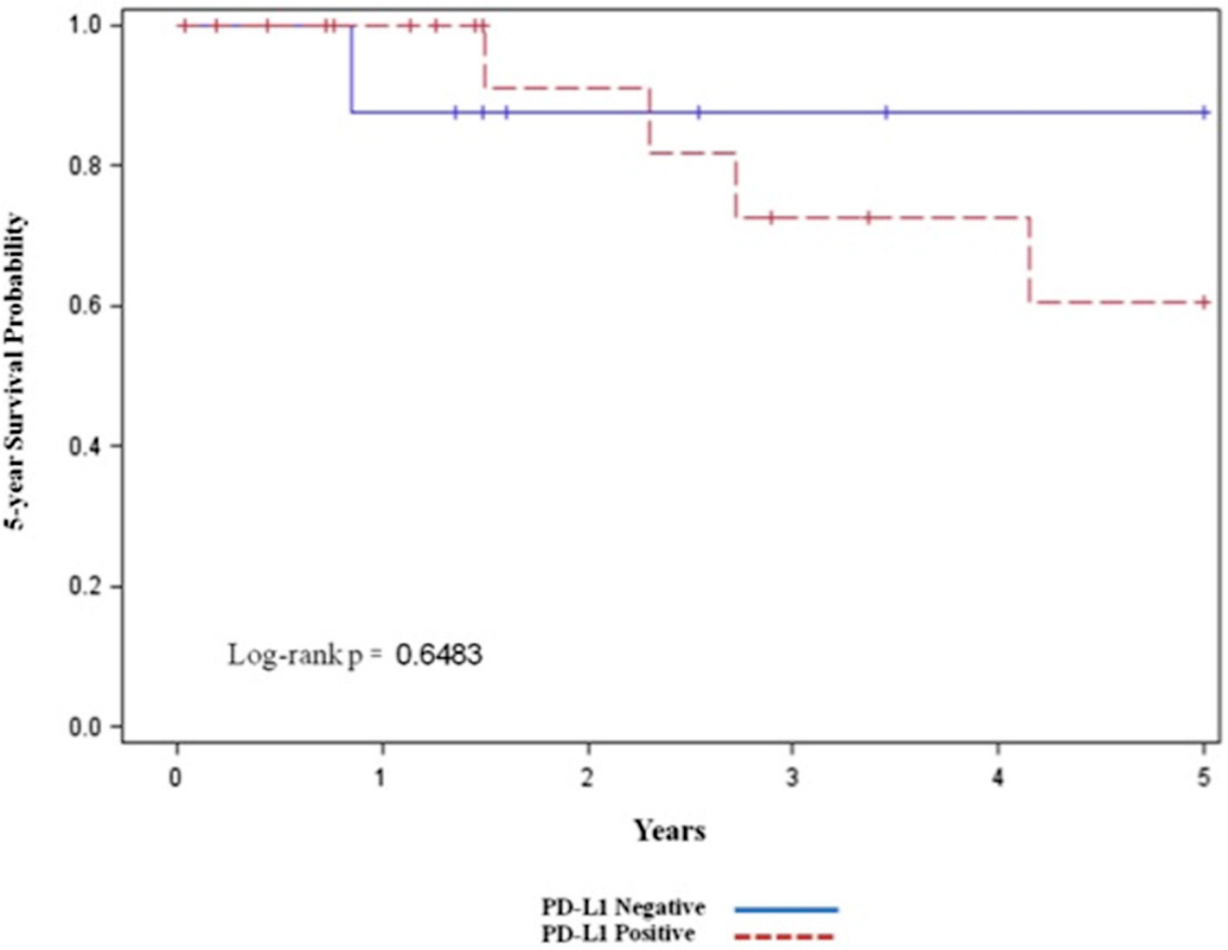
**PD-L1 expression on TIMC and 5-Year survival rate (univariate analysis).**

Overall, 6 patients died during the follow up period. Positive PD-L1 expression in tumor cell membrane was not associated with 5-year survival (univariate analysis; two-sided *p* = 0.65) (Figure 2).

## Discussion

Multiple retrospective analyses described the correlation between levels of PD-L1 expression and prognosis in several malignancies [20]. Some of these studies in renal cell carcinoma (RCC), breast cancer, and non-small cell lung cancer (NSCLC), demonstrated that higher levels of PD-L1 expression in tumor cells were associated with an unfavorable prognosis [24-26]. In contrast, higher PD-L1 expression on immune cells was associated with longer OS in melanoma and metastatic urothelial carcinoma [27,28]. To our knowledge, this is the first study to characterize PD-L1 expression and its clinical significance in ACC.

Tumor stage at diagnosis is the most important prognostic factor in ACC [29]. However, different clinical courses have been described among patients into the same tumor stage. Some retrospective analyses have reported that functional tumors may be associated with worse prognosis [30]. In addition, few studies have established histological or molecular markers, such as Ki67 index or *TP53* mutations, as predictors of poor prognosis and its value still needs to be confirmed [31]. From a clinician perspective, to investigate biomarkers that can predict response to treatments may be important in the decision-making process in the era of personalized medicine. In our analysis, PD-L1 positivity was observed in approximately 11% of ACC cases and did not correlate with stage at diagnosis (UICC or ENSAT), grade, and excessive secretion of hormones. Furthermore, no correlations were found between PD-L1 expression and survival at 5 years.

Some tumors are infiltrated by immune cells and it can dynamically influence the host immune response against tumor [32]. Interestingly, Willenberg and colleagues provided evidence of the involvement of immune cells and interleukin-2 (IL-2) cytokine stimulation in the formation of an adrenocortical tumor in a patient with Cushing’s syndrome [33]. While little is known about the immune microenvironment in ACC, these findings may open new avenues on the understanding of tumor biology and development of new treatment strategies. The interaction between PD-1 and its ligand PD-L1 limits T cell activation in response to certain antigens in order to prevent immune-mediated damage in healthy tissue. Furthermore, chronic antigen exposure increases the levels of PD-L1 expression, resulting in T cell “exhaustion” and reduced immune control of tumor progression [34]. Tumor cells have the ability to express PD-L1 as an adaptive mechanism of resistance that can evade the immune system, resulting in tumor growth and more aggressive disease.

With the goal of restoring effective T cell responses, the inhibition of immune checkpoints such as PD-1 or PD-L1 has been considered attractive therapeutic targets using monoclonal antibodies. A set of well conducted clinical trials have reported encouraging clinical activity on PD-1/PD-L1 blockade across multiple tumor types. The first phase I clinical trial of nivolumab, an anti-PD-1 monoclonal antibody, showed significant clinical activity in RCC, melanoma, and NSCLC, leading to deeper investigations [35]. Other agents targeting this pathway have supported these early results [36]. In addition, combinations of immunomodulatory agents have been tested in different solid tumors and reported promising results [37].

No biomarkers have been established to precisely select patients for therapeutic strategies blocking the PD-1/PD-L1 axis. Moreover, while several studies have reported that PD-L1 expression in both tumor cell or tumor infiltrating immune cells is a potential predictor of response to immunomodulatory agents, the meaning and significance of PD-L1 expression in tumor cells or immune cells is still being investigated [20]. Preliminary results from a phase I study of an anti-PD-L1 inhibitor (MPDL3280A) in patients with advanced urothelial carcinoma showed response rates of 52% in patients with PD-L1 positive in immune cells vs. 14% in PD-L1 negative patients [38]. Interestingly, accumulating evidence shows that durable responses can also occur in patients who do not express PD-L1 on tumor cell membrane and/or tumor infiltrating immune cells [39]. This raises important considerations including tumor heterogeneity and tumor microenvironment alterations that need to be investigated in further studies.

Though this is the first study to date to evaluate the prevalence and prognostic significance of PD-L1 expression in ACC, there are several limitations. First, the retrospective nature of this analysis has led to missing data which may result in selection bias. In addition, considering that ACC incidence is low, it is difficult to perform studies with large and homogeneous cohorts. In our study, the sample size and the number of events in each group according to PD-L1 positivity were very small, limiting our ability to detect statistically significant differences between groups. At the same time, there may be a missing-variable bias, since other clinico-pathological features such as Ki67 were not available for the majority of patients in this cohort. Furthermore, tumor heterogeneity is a confounding factor and differences between of primary and metastasis still need to be investigated. In 2 patients from our series, we had the opportunity to review primary and metastatic sites of disease. In one case, the primary tumor and one met were evaluated; in the other case the primary tumor plus 3 independent metastases were compared. Interestingly, in both cases the same results regarding PD-L1 expression (positive or negative) were observed in primary and metastatic samples stained for PD-L1. In this study, we tried to minimize the selection bias due to tumor heterogeneity by analyzing whole tissue sections from surgical resections and excluding needle biopsies*.* Finally, we focused on the clinical significance of PD-L1 expression in either, tumor cells and immune cells. However, the criteria defining PD-L1 positivity (cut-offs) as well as the antibodies used to stain for PD-L1 have varied among different studies. Therefore, comparisons with other studies should be done with caution and a more specific definition of immune cells subtypes should be focus of further studies. Although the same methodology used in this analysis has been applied to other studies, the methodology for PD-L1 staining should be standardized to allow for reliable evaluation of PD-L1 expression and comparison among differing cohorts [40].

## Conclusions

In summary, ACC can express PD-L1 on both tumor cell membrane and immune cells and it may represent a potential target for therapeutic interventions. The current progress in cancer immunotherapy warrants prospective validation of our findings and further investigation of agents blocking the PD-1/PD-L1 pathway in this aggressive disease.

## Methods

### Patients and samples

Twenty-eight patients with ACC treated surgically at Dana-Farber Cancer Institute or Brigham and Women’s Hospital were retrospectively selected. Formalin fixed paraffin-embedded (FFPE) blocks from primary tumors or metastases were retrieved, and for each patient one representative tumor block was selected for analysis by a genitourinary pathologist. Baseline clinico-pathological characteristics such as age, gender, tumor size, grade, stage using UICC and ENSAT staging systems, hormone-related symptoms at clinical presentation, as well as follow up data were retrospectively collected for all patients. Patients who presented with Cushing’s syndrome, virilization, or feminilization were classified as functional tumors. This study received Institutional Review Board approval before tumor staining and data acquisition.

### Immunohistochemistry

PD-L1 expression was evaluated by immunohistochemistry (IHC) using a mouse monoclonal anti-PD-L1 antibody (405.9A11) developed in Dr. Gordon Freeman’s laboratory at Dana-Farber Cancer Institute. The immunohistochemical assay was validated using FFPE cell line controls known to be positive or negative for PD-L1 expression by flow cytometry [41]. Four micron-thick tumor sections were stained with an anti-PD-L1 antibody at a concentration of 3.25 ug/ml on a Benchmark XT autostainer (Ventana Medical System, Tucson, AZ) with standard antigen retrieval (CC1 buffer, pH8.0, #950-124, Ventana). UltraView Universal DAB Detection kit (#760-500, Ventana) was used according to the manufacturer’s instruction. Counterstaining was performed as part of the automated staining protocol using hematoxylin (#760-2021, Ventana). After staining, slides were then washed in soap water and distilled water, dehydrated in graded alcohol and xylene, mounted, and cover slipped.

CD45 immunostaining was performed on adjacent four micron-thick tumor sections, which were initially deparaffinized, rehydrated and heated with a pressure cooker to 125°C for 30 seconds in citrate buffer for antigen retrieval and then incubated with peroxidase (Dako #S2003, Carpinteria, CA) and protein blocking reagents (Dako #X0909) each for 5 minutes. Sections were then incubated with anti-CD45 (1:100, Dako, clone 2B11 + PD7/26) antibody for 1 hour at room temperature followed by incubation with the Dako EnVision + System HRP labeled polymer anti-mouse (Dako #K4001) for 30 minutes. All sections were developed using the DAB chromogen kit (Dako K3468) for 2 minutes and then lightly counterstained with hematoxylin.

### Scoring of PD-L1 expression on tumor cell membrane

For each sample, the percentage of tumor cells with PD-L1 expression on the cell membrane was estimated by two independent genitourinary pathologists who were blinded to clinical outcomes.

### Scoring of PD-L1 expression in Tumor Infiltrating Mononuclear Cells (TIMC)

TIMC were identified on the basis of IHC positivity for CD45, a pan-leukocyte marker expressed in lymphocytes, macrophages and dendritic cells [42,43]. The extent of TIMC was recorded as absent (0), focal (1), mild (2), moderate (3) and marked (4). The percentage of PD-L1 expression in TIMC was evaluated semi-quantitatively according to three categories: 0% = 0, <5% = 1, and ≥5% = 2. An adjusted score was then calculated multiplying the percentage of TIMC that stained positive for PD-L1 and the extent of infiltrating immune cells, as previously reported [44]. Staining for PD-L1 was not performed in one patient in whom the available specimen was from a lymph node given that tumoral and non-tumoral immune cells could not be distinguished.

### Statistical analysis

In this exploratory biomarker study, the pre-defined primary objective of this study was to characterize levels of PD-L1 expression on tumor cell membrane and TIMC in patients with ACC. Secondary endpoints were the correlation of PD-L1 expression with clinico-pathological features as well as 5-year survival rates. Five-year survival rate was defined as the time period between date of diagnosis and the date of death, or censored at 5 years after diagnosis. PD-L1 tumor positivity on tumor cells was defined as ≥5% tumor cell membrane staining. For PD-L1 expression in TIMC, any score greater than zero was considered positive.

Statistical analyses were performed using SAS (version 9.2; SAS Institute Inc., Cary, NC, USA). Descriptive data are presented as mean and standard deviation (SD), or percentage. Comparisons between PD-L1 expression and clinico-pathological features were evaluated using Fisher’s exact test for categorical variables and unpaired t-test for continuous variables. Kaplan-Meier method estimated the distribution of 5-year survival rates by PD-L1 positivity. The association of 5-year survival rates with PD-L1 expression (negative vs. positive) was assessed by log-rank test and univariate Cox proportional regression analysis. Multivariate analysis were to be performed only if p-values were <0.05, considered statistically significant.

## Abbreviations

ACC: Adrenocortical carcinoma
EGFR: Epidermal growth factor receptor
ENSAT: European network for the study of adrenal tumors
FDFR: Fibroblast growth factor receptor
FFPE: Formalin fixed paraffin-embedded
FIRM-ACT: First international randomized trial in locally advanced and metastatic ACC treatment
IGF-1R: Insulin-like growth factor 1 receptor
IHC: Immunohistochemistry
IL-2: Interleukin-2
M/EDP: Mitotane, etoposide, doxorubicin, and cisplatin
mTOR: Mammalian target of rapamycin
NSCLC: Non-small cell lung cancer
OS: Overall survival
PD-1: Programmed death-1
PD-L1: Programmed death ligand-1
PFS: Progression-free survival
RCC: Renal cell carcinoma
SD: Standard deviation
SM: Mitotane and streptozocin
TIMC: Tumor infiltrating mononuclear cells
UICC: International Union Against Cancer
VEGF: Vascular endothelial growth factor

## References

[1] Third national cancer survey: incidence data. Natl Cancer Inst Monogr. 1975;41:i-x, 1–454. 1165769

[2] Kebebew E, Reiff E, Duh QY, Clark OH, McMillan A (2006). Extent of disease at presentation and outcome for adrenocortical carcinoma: have we made progress?. World J Surg.

[3] Bilimoria KY, Shen WT, Elaraj D, Bentrem DJ, Winchester DJ, Kebebew E (2008). Adrenocortical carcinoma in the United States: treatment utilization and prognostic factors. Cancer.

[4] Khorram-Manesh A, Ahlman H, Jansson S, Wangberg B, Nilsson O, Jakobsson CE (1998). Adrenocortical carcinoma: surgery and mitotane for treatment and steroid profiles for follow-up. World J Surg.

[5] Luton JP, Cerdas S, Billaud L, Thomas G, Guilhaume B, Bertagna X (1990). Clinical features of adrenocortical carcinoma, prognostic factors, and the effect of mitotane therapy. N Engl J Med.

[6] op den Winkel J, Pfannschmidt J, Muley T, Grunewald C, Dienemann H, Fassnacht M (2011). Metastatic adrenocortical carcinoma: results of 56 pulmonary metastasectomies in 24 patients. Ann Thorac Surg.

[7] Fay AP, Elfiky A, Telo GH, McKay RR, Kaymakcalan M, Nguyen PL, et al. Adrenocortical carcinoma: the management of metastatic disease. Crit Rev Oncol Hematol. 2014. doi:10.1016/j.critrevonc.2014.05.009. 10.1016/j.critrevonc.2014.05.009 PMC4578298 24958272

[8] Fassnacht M, Terzolo M, Allolio B, Baudin E, Haak H, Berruti A (2012). Combination chemotherapy in advanced adrenocortical carcinoma. N Engl J Med.

[9] Berruti A, Terzolo M, Pia A, Angeli A, Dogliotti L (1998). Mitotane associated with etoposide, doxorubicin, and cisplatin in the treatment of advanced adrenocortical carcinoma. Italian Group for the study of adrenal cancer. Cancer.

[10] Khan TS, Imam H, Juhlin C, Skogseid B, Grondal S, Tibblin S (2000). Streptozocin and o, p’DDD in the treatment of adrenocortical cancer patients: long-term survival in its adjuvant use. Ann Oncol.

[11] Sperone P, Ferrero A, Daffara F, Priola A, Zaggia B, Volante M (2010). Gemcitabine plus metronomic 5-fluorouracil or capecitabine as a second-/third-line chemotherapy in advanced adrenocortical carcinoma: a multicenter phase II study. Endocr Relat Cancer.

[12] Barlaskar FM, Spalding AC, Heaton JH, Kuick R, Kim AC, Thomas DG (2009). Preclinical targeting of the type I insulin-like growth factor receptor in adrenocortical carcinoma. J Clin Endocrinol Metab.

[13] De Martino MC, van Koetsveld PM, Feelders RA, Sprij-Mooij D, Waaijers M, Lamberts SW (2012). The role of mTOR inhibitors in the inhibition of growth and cortisol secretion in human adrenocortical carcinoma cells. Endocr Relat Cancer.

[14] Carden ESK CP, Jones RL, Alam SM, Johnson FM, Stephens AW, Poondru S (2010). Phase I study of intermittent dosing of OSI-906, a dual tyrosine kinase inhibitor of insulin-like growth factor-1 receptor (IGF- 1R) and insulin receptor (IR) in patients with advanced solid tumors. J Clin Oncol.

[15] Kroiss M, Quinkler M, Johanssen S, van Erp NP, Lankheet N, Pollinger A (2012). Sunitinib in refractory adrenocortical carcinoma: a phase II, single-arm, open-label trial. J Clin Endocrinol Metab.

[16] Jimenez P, Guix M, Milagro NL, Mateos LL, Mendez Vidal MJ, Climent Duran MA, et al. Phase II study of dovitinib in first-line metastatic or nonresectable primary adrenocortical carcinoma (ACC): SOGUG study 2011–03. J Clin Oncol. 2012;30(suppl; abstr TPS4688):2014.

[17] O’Sullivan C, Edgerly M, Velarde M, Wilkerson J, Venkatesan AM, Pittaluga S (2014). The VEGF inhibitor axitinib has limited effectiveness as a therapy for adrenocortical cancer. J Clin Endocrinol Metab.

[18] Lerario AM, Worden FP, Ramm CA, Hasseltine EA, Stadler WM, Else T (2014). The combination of insulin-like growth factor receptor 1 (IGF1R) antibody cixutumumab and mitotane as a first-line therapy for patients with recurrent/metastatic adrenocortical carcinoma: a multi-institutional NCI-sponsored trial. Hormones Cancer.

[19] Francisco LM, Sage PT, Sharpe AH (2010). The PD-1 pathway in tolerance and autoimmunity. Immunol Rev.

[20] McDermott DF, Atkins MB (2013). PD-1 as a potential target in cancer therapy. Cancer Med.

[21] Thompson RH, Dong H, Kwon ED (2007). Implications of B7-H1 expression in clear cell carcinoma of the kidney for prognostication and therapy. Clin Cancer Res.

[22] Ohigashi Y, Sho M, Yamada Y, Tsurui Y, Hamada K, Ikeda N (2005). Clinical significance of programmed death-1 ligand-1 and programmed death-1 ligand-2 expression in human esophageal cancer. Clin Cancer Res.

[23] Lughezzani G, Sun M, Perrotte P, Jeldres C, Alasker A, Isbarn H (2010). The European Network for the study of adrenal tumors staging system is prognostically superior to the international union against cancer-staging system: a North American validation. Eur J Cancer.

[24] Thompson RH, Kwon ED (2006). Significance of B7-H1 overexpression in kidney cancer. Clin Genitourin Cancer.

[25] Thompson RH, Gillett MD, Cheville JC, Lohse CM, Dong H, Webster WS (2004). Costimulatory B7-H1 in renal cell carcinoma patients: Indicator of tumor aggressiveness and potential therapeutic target. Proc Natl Acad Sci U S A.

[26] Boland JM, Kwon ED, Harrington SM, Wampfler JA, Tang H, Yang P (2013). Tumor B7-H1 and B7-H3 expression in squamous cell carcinoma of the lung. Clin Lung Cancer.

[27] Mullane SA, Werner L, Callea M, Fay AP, Leow JJ, Choueiri TK (2014). PD-L1 expression in mononuclear cells and not in tumor cells, correlated with prognosis in metastatic urothelial carcinoma. J Clin Oncol.

[28] Harshman LC, Choueiri TK, Drake C, Stephen Hodi F (2014). Subverting the B7-H1/PD-1 pathway in advanced melanoma and kidney cancer. Cancer J.

[29] Karakousis CP, Rao U, Moore R (1985). Adrenal adenocarcinomas: histologic grading and survival. J Surg Oncol.

[30] Hogan TF, Gilchrist KW, Westring DW, Citrin DL (1980). A clinical and pathological study of adrenocortical carcinoma: therapeutic implications. Cancer.

[31] Assie G, Antoni G, Tissier F, Caillou B, Abiven G, Gicquel C (2007). Prognostic parameters of metastatic adrenocortical carcinoma. J Clin Endocrinol Metab.

[32] Mantovani A, Romero P, Palucka AK, Marincola FM (2008). Tumour immunity: effector response to tumour and role of the microenvironment. Lancet.

[33] Willenberg HS, Stratakis CA, Marx C, Ehrhart-Bornstein M, Chrousos GP, Bornstein SR (1998). Aberrant interleukin-1 receptors in a cortisol-secreting adrenal adenoma causing cushing’s syndrome. N Engl J Med.

[34] Quezada SA, Peggs KS (2013). Exploiting CTLA-4, PD-1 and PD-L1 to reactivate the host immune response against cancer. Br J Cancer.

[35] Topalian SL, Hodi FS, Brahmer JR, Gettinger SN, Smith DC, McDermott DF (2012). Safety, activity, and immune correlates of anti-PD-1 antibody in cancer. N Engl J Med.

[36] DC Cho, JA Sosman, M Sznol, MS Gordon, A Hollebecque, O Hamid, et al. Clinical activity, safety, and biomarkers of MPDL3280A, an engineered PD-L1 antibody in patients with metastatic renal cell carcinoma (mRCC). ASCO Annual Meeting. J Clin Oncol. 2013;31(suppl; abstr 4505).

[37] Wolchok JD, Kluger H, Callahan MK, Postow MA, Rizvi NA, Lesokhin AM (2013). Nivolumab plus ipilimumab in advanced melanoma. N Engl J Med.

[38] Bellmunt J, Powles T, Braiteh F, Vogelzang N, Cruz C, Burris H (2014). Inhibition of PD-L1 by MPDL3280A leads to clinical activity in pts with metastatic urothelial bladder cancer (UBC). Ann Oncol.

[39] Choueiri TK, Fishman MN, Escudier B, Kim JJ, Kluger H, Stadler WM (2014). Immunomodulatory activity of nivolumab in previously treated and untreated metastatic renal cell carcinoma (mRCC): biomarker-based results from a randomized clinical trial. J Clin Oncol.

[40] Choueiri TK. Highlights of the Day III Session - Genitourinary (Nonprostate) Cancer 2014

[41] Green MR, Monti S, Rodig SJ, Juszczynski P, Currie T, O’Donnell E (2010). Integrative analysis reveals selective 9p24.1 amplification, increased PD-1 ligand expression, and further induction via JAK2 in nodular sclerosing Hodgkin lymphoma and primary mediastinal large B-cell lymphoma. Blood.

[42] Hsieh C, Chang A, Brandt D, Guttikonda R, Utset TO, Clark MR (2011). Predicting outcomes of lupus nephritis with tubulointerstitial inflammation and scarring. Arthritis Care Res.

[43] Haley KJ, Sunday ME, Wiggs BR, Kozakewich HP, Reilly JJ, Mentzer SJ (1998). Inflammatory cell distribution within and along asthmatic airways. Am J Respir Crit Care Med.

[44] Choueiri TK, Fay AP, Gray KP, Callea M, Ho TH, Albiges L (2014). PD-L1 expression in nonclear-cell renal cell carcinoma. Ann Oncol.

